# Multi-dimensional frailty and Oral Health-Related Quality-of-Life in older adults: a cross-sectional study

**DOI:** 10.1007/s11136-025-03969-0

**Published:** 2025-05-07

**Authors:** Ana Cristina Mafla, Mauricio Herrera-López, Bruno Gutiérrez-Quiceno, Nico De Witte, Falk Schwendicke

**Affiliations:** 1 School of Dentistry, Universidad Cooperativa de Colombia, Pasto, Nariño, Colombia; 2 Department of Psychology, Universidad de Nariño, Pasto, Nariño, Colombia; 3 Faculty of Health, School of Dentistry, Universidad del Valle, Cali, Valle del Cauca, Colombia; 4 Faculty of Psychology and Educational Science, Vrije Universiteit Brussel, University College Ghent, Brussels, Ghent, Belgium; 5 Department of Conservative Dentistry and Periodontology, Ludwig-Maximilians- Universität, München, Germany

**Keywords:** Older adults, Oral health, Frailty, Psychology, Colombia

## Abstract

**Background:**

Frailty is an age-associated decline of physiological functions, with psychological, social and environmental dimensions. Frailty is associated with oral health and well-being. We aimed to determine the association between Oral Health-Related Quality-of-Life (OHRQoL) and multi-dimensional frailty in older Colombians.

**Methods:**

An observational study was conducted and included 1,079 older adults aged 60 to 105 years from Pasto, Colombia, recruited from different government programs targeting older adults. Socio-demographic characteristics were registered; OHRQoL was determined using the self-administered Colombian Geriatric Oral Health Assessment Index (GOHAI) version, and frailty using the Comprehensive Frailty Assessment Instrument (CFAI) and covariates identified with directed acyclic graph (DAG) analysis. A descriptive analysis and a generalized linear model (GLM) were performed.

**Results:**

The sample was comprised of 386 males (35.8%) and 693 (64.2%) females. The mean age was 71.85 years (standard deviation, SD=8.55). The mean GOHAI was 49.60 (SD=8.91). GLM found that overall frailty dimensions of the CFAI negatively impacted OHRQoL with psychological dimension having the largest impact (β= -0.44, 95% CI:-0.54– -0.34, P<0.001).

**Conclusion:**

Comprehensive frailty assessment allows us to map various dimensions of frailty and the impact they have on OHRQoL.

**Supplementary Information:**

The online version contains supplementary material available at 10.1007/s11136-025-03969-0.

## Background

“Frailty is theoretically defined as a clinically recognizable state of increased vulnerability resulting from aging-associated decline in functions across multiple physiological systems.” [1] The global prevalence of frailty has been reported as 3–27% [2]. The highest prevalence of frailty was found in nursing-home residents, hospitalized older adults and community dwellers [3]. Data for Colombia show prevalence to be at the higher end of the spectrum, with a particularly high prevalence in indigenous people [4]. Frailty has been associated with high age, living alone and without a caregiver, hospitalizations or falls [5], and a range of medical conditions [6–8].

Older individuals have been shown to suffer from increasing risks of periodontitis [9] and dental caries, likely related to poor oral hygiene as a result of limited fine motor skills [10], and low salivary flow rate and quality, resulting in decreased levels of antimicrobial agents and calcium [11]. Both periodontitis and dental caries as well as their sequel, tooth loss, have been associated with quality of life [12], as they determine mastication efficiency and hence dietary options, but also phonetics and aesthetics [13].

Oral health and Oral Health-Related Quality-of-Life (OHRQoL) have been found to be associated with frailty in several studies, likely via oral pain, chewing problems and the resulting weight loss [14–16]. Notably, most data on this association describe frailty as unidimensional, while frailty has been shown to vary across different genders, socioeconomic and educational strata, among others [17]. Reflecting not only the physical aspects related to frailty but also the social and environmental risk factors of both frailty and low OHRQoL [18–19], may allow to more comprehensively characterize the relationship between oral health and frailty. The objective of the present study was to determine the association between OHRQoL and frailty, captured via a comprehensive multi-dimensional measure, in older Colombians. We hypothesized that the psychological dimension of frailty would have a higher impact on OHRQoL.

## Methods

### Study design and sample

**Fig. 1:**
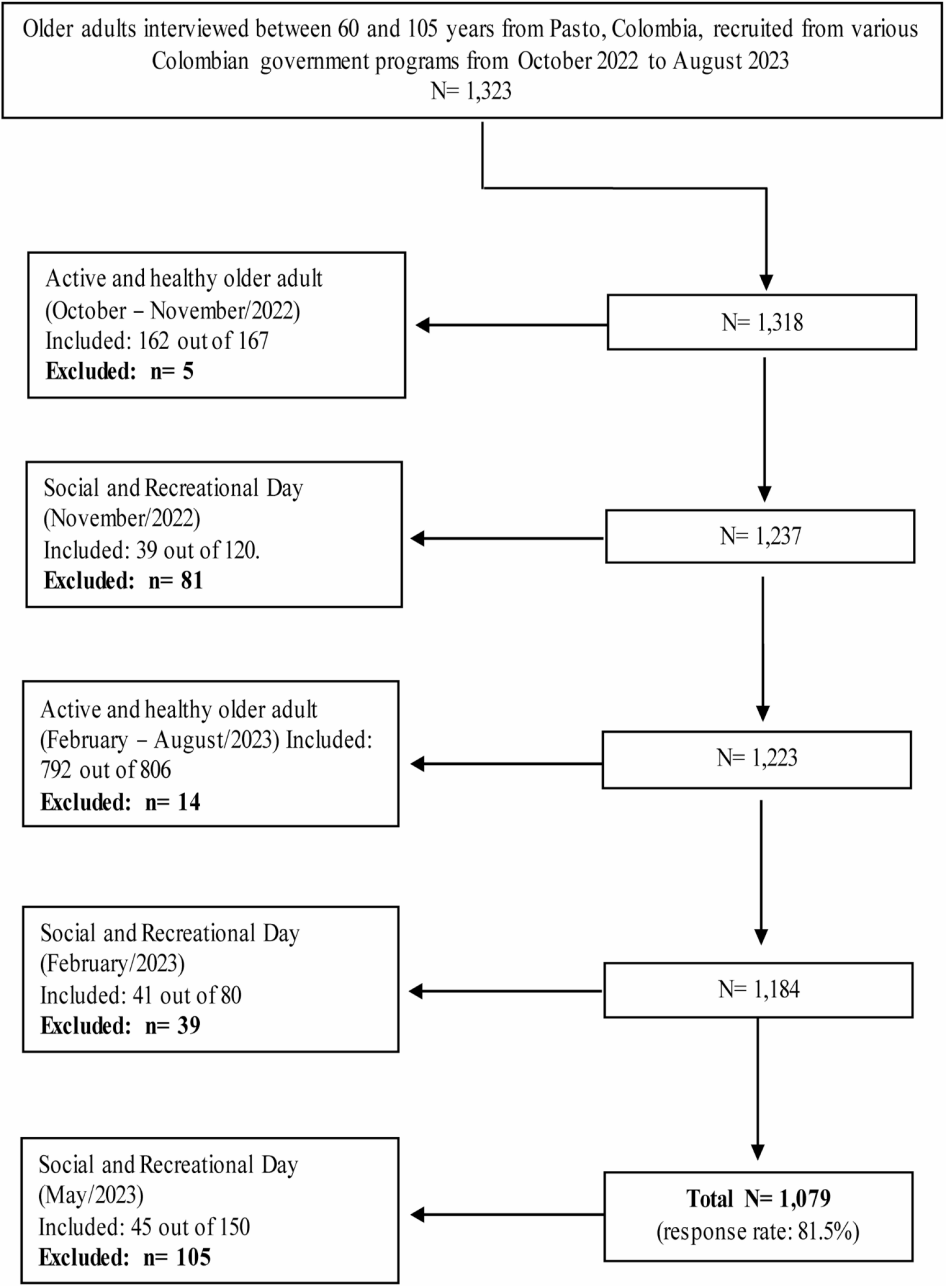
Flow chart of the recruited sample of older adults ≥ 60 years old

A cross-sectional study was conducted including a non-probabilistic sample of 1,079 individuals aged 60 to 105 years from Pasto, Colombia, who were recruited from various Colombian government programs targeting older adults (Fig. 1). Data was collected from October 2022 to August 2023. Voluntary participants, male and female ≥ 60 years old, being able to answer the survey (e.g. absence of Alzheimer´s disease, delirium, dementia, depression, anxiety disorders, severe urinary incontinence and severe pain preventing them to sit for sufficiently long time) were included.

### Measures

Through a series of 30–40 min interviews to older adults we collected different sociodemographic characteristics as follows: age (number of years); biological sex according to World Health Organization [20] (classified as male/female); place of birth (catalogued as Pasto, capital city/other place); residency (categorized as Pasto, capital city/other place); living zone (coded as rural/urban); socioeconomic status (SES) grouped according to housing quality indicators set by the Colombian government [21] (classified in two groups as low/middle and high due to the strata distribution in the locality); marital status: having a wife/husband/partner (categorized as yes/no); education (classified as none/primary school/high school/university); occupation (coded as yes and no); having a health insurance (classified as yes/no); type of health insurance (catalogued as public/private); and barriers to oral health care categorized as “accessibility” (getting an appointment, inability to get preventive services, unavailable transportation, too high distance to oral health clinics or difficulty to access to dental clinic office), economic stress (high costs of dental treatments and personal loans or other debts) and past dental experience (dental fear, bad past dental experiences and little awareness of the importance of dental check-ups). Additionally, further aspects related to oral health such as last dental visit (more than 5 years ago, 1–5 years ago and less than 1 year ago); presence of natural teeth (categorized as no natural teeth, 1–10 natural teeth, 11–21 natural teeth and > 21 natural teeth) and dental prosthetics being used ( “no, I have natural teeth”, “yes, for the upper jaw”, “yes, for the lower teeth”, “yes, for the upper and lower teeth”, and “I do not have teeth but I do not use my denture”) were asked.

### Instruments

OHRQoL was evaluated with the Geriatric Oral Health Assessment Index (GOHAI) and frailty with the Comprehensive Frailty Assessment Instrument (CFAI), both reported as continuous variables.

### Geriatric oral health assessment index (GOHAI)

The GOHAI [22] is a 12-item self-reported instrument that measures oral functions and problems, the psychosocial impacts derived from oral diseases, and pain and discomfort [23] via Likert scales that range from 1 (“always”) to 5 (“never”). The items 1, 2, 4, 6, 8, 9, 10, 11 and 12 are scored from 1 to 5, and items 3, 5 and 7 are scored reversely. The total sum of all 12 items (ranging from 12 to 60) is then formed, with a higher sum indicating fewer problems. The simple count score (SC-GOHAI) is obtained by summing up the scores of each answer and has been shown to have robust good psychometric properties in different languages [24–28]. A Colombian GOHAI version, which has been demonstrated to be a valid and reliable tool in this population, was used. Values between 51 and 56 indicated moderate and ≤ 50 a low OHRQoL [29]. Permission to use this scale has been granted by the author of Spanish GOHAI version.

### Comprehensive frailty assessment instrument (CFAI)

The CFAI [30] is a self-administered instrument developed to detect frailty in the community, using a multidimensional perspective that assesses physiological, psychological, social, and environmental domains. The physical domain reflects limitations in physical activities due to health problems; the psychological domain incorporates mood disorders and emotional loneliness; the social domain includes social loneliness and having a social support network (including family, other relatives, friends, neighbors and acquaintances); and the environmental domain reflects on housing and environmental conditions. Items were scored on 3, 4 and 5-point Likert scales, ranging from “not at all to more than 3 months” (physical dimension); “not at all to considerably more than usual” and “I completely disagree to I completely agree” (psychological dimension); “I completely disagree to I completely agree” (social dimension) and “not applicable at all to completely applicable” (environmental dimension). Values > 21.9 indicate mild and > 38.8 high frailty, respectively [31]. Higher scores demonstrated more frailty elements. Permission to use this scale has been granted by the author.

### Statistical analysis

Descriptive statistics were calculated to report sociodemographic characteristics, the GOHAI sum score and the dimensions scores of the CFAI. Cronbach’s alpha (α) was used to measure the internal consistency of the scale CFAI (α = 0.81) and GOHAI (α = 0.86). A non-parametric Chi square (χ^2^) test was used to determine independence between and among groups, and as these variables were not normally distributed, Mann-Whitney-U-test and Kruskal-Wallis-test were utilized to compare variables.

**Fig. 2:**
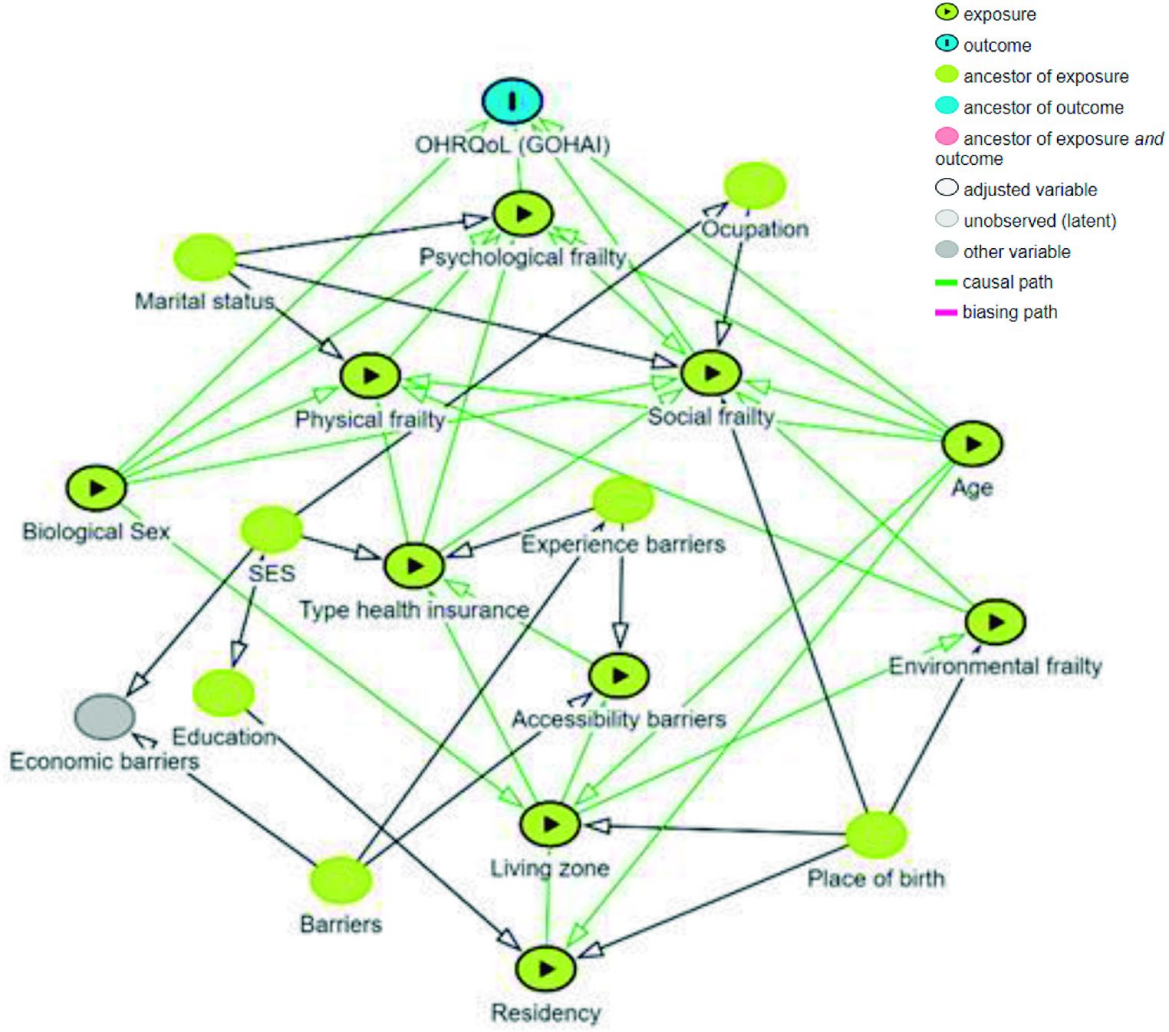
Directed acyclic graph (DAG) model for Oral Health-Related Quality-of-Life (OHRQoL) via GOHAI. The analysis shows causal paths of exposures to the outcome

To evaluate the causal effect of the exposure of potential variables including confounders or mediators on the outcome, a directed acyclic graph (DAG) model was first developed. The browser-based software DAGitty v 3.1 [32] was used for modeling causal relationships among variables (Fig. 2). A generalized linear model (GLM) through a simple linear regression was estimated for a non-normal distribution of the main outcome to determine the associations between OHRQoL (via GOHAI) and sociodemographic characteristics, and comprehensive frailty (via CFAI) following the causal path obtained from DAG analysis. Data were totally completed by respondents in controlled interviews, for that reason no data were missing. Statistical analyses were performed using statistical software SPSS for Windows, Version 29.0 (IBM, Armonk, USA). The significance level was set at *P* < 0.05.

### Bioethics statement

The protocol of the study was approved by the Bioethics Committee of Universidad Cooperativa de Colombia (BIO321, 10/26/2022). The study was conducted in accordance with the Helsinki principles [33]. All older adults provided their written informed consent to participate in this investigation. The study reporting is in accordance with the Strengthening the Reporting of Observational Studies in Epidemiology (STROBE) statement (Annex-1) [34].

## Results

The sample was comprised of 386 males (35.8%) and 693 (64.2%) females. The mean age was 71.85 years (standard deviation (*SD*) = 8.55). Table 1 displays the characteristics of the sample. There was a higher frequency of working females (72.4%) than males (51.3%), and females also showed more barriers to oral healthcare than males.

**Table 1:** Sociodemographic characteristics of 1,079 older adults, from Pasto, Colombia

Variable	Males *(* *n* * = 386)*		Females *(n* * = 693)*		Total *(n = 1*,*079)*		*P*-value^a^
	*n*	%	*n*	%	*n*	%	
**Group of age**							0.488
60–74 years	232	60.1	442	63.8	674	62.5	
75–84 years	122	31.6	198	28.6	320	29.6	
≥ 85 years	32	8.3	53	7.6	85	7.9	
**Place of birth**							0.547
Pasto (capital city)	204	52.8	353	50.9	557	51.6	
Other place	182	47.2	340	49.1	522	48.4	
**Residency**							0.921
Pasto (capital city)	330	85.5	594	85.7	924	85.6	
Other place	56	14.5	99	14.3	155	14.4	
**Living zone**							0.020
Rural	62	16.1	77	11.1	139	12.9	
Urban	324	83.9	616	88.9	940	87.1	
**Socioeconomic status**							0.003
Low	238	61.7	489	70.6	727	67.4	
Middle/High	148	38.3	204	29.4	352	32.6	
**Marital status** (wife/husband/partner)							< 0.001
Yes	193	50.0	231	33.3	424	39.3	
No	193	50.0	462	67.7	655	60.7	
**Health insurance** (having an insurance)							-
Yes	386	100.0	693	100.0	1.079	100.0	
No	-	-	-	-	-	-	
**Type of health insurance**							0.090
Public	235	60.9	385	55.6	620	57.5	
Private	151	39.1	308	44.4	459	42.5	
**Education**							0.004
None	36	9.3	88	12.7	124	11.5	
Primary	154	39.9	329	47.5	483	44.8	
Secondary	128	32.2	190	27.4	318	29.5	
University	68	17.6	86	12.4	154	14.3	
**Occupation**							< 0.001
Yes	198	51.3	502	72.4	700	64.9	
No	188	48.7	191	27.6	379	35.1	
**Barriers to oral healthcare**							< 0.001
0–1 barriers	95	24.6	89	12.8	184	17.1	
2–3 barriers	179	46.4	361	52.1	540	50.0	
≥ 4 barriers	112	29.0	243	35.1	355	32.9	
**Accessibility barriers**							0.002
Yes	213	55.2	449	64.8	662	61.4	
No	173	44.8	244	35.2	417	38.6	
**Economic barriers**							< 0.001
Yes	258	66.8	551	79.5	809	75.0	
No	128	32.2	142	20.5	270	25.0	
**Experience barriers**							0.692
Yes	273	70.7	498	71.9	771	71.5	
No	113	29.3	195	28.1	308	28.5	

Note: ^a^Independence tests derived from χ^2^

Regarding dental check-ups, 403 (37.3%) older adults had their last dental visit > 5 years ago, being more frequent in participants ≥ 85 years old (62, 72.9%) (*P* < 0.001). Overall, 481 (44.6%) have no teeth, 324 (30.0%) had between 1 and 10 teeth, 167 (15.5%) had between 11 and 21% and 107 (9.9%) had > 21 teeth. According to dental prosthetics use, 320 (29.7%) utilized one for replacing the upper teeth, 49 (4.5%) used for replacing lower teeth, 515 (47.7%) used for replacing both, upper and lower teeth, and 102 (9.5%) had no teeth but they did not use dental prosthetics.

The mean GOHAI was 49.60, SD = 8.91. Table 2 exhibits the OHRQoL by sociodemographic strata. GOHAI differed along living zones, type of health insurance and socioeconomic status, with those living in the rural zone (mean = 46.29, SD = 9.11), having public health insurance (mean = 47.71, SD = 8.92) and having low SES (mean = 48.61, SD = 8.95) showing significantly lower scores, i.e. lower OHRQoL.

**Table 2:** Means and standard deviations (SD) of geriatric oral health assessment index (GOHAI) by sociodemographic characteristics

Variable	Total *(n = 1*,*079)*		mean	SD	*P*-value
	*n*	%			
**Group of age**					< 0.001^a^
60–74 years	674	62.5	48.62	9.27	
75–84 years	320	29.6	51.31	8.05	
≥ 85 years	85	7.9	50.89	8.00	
**Sex**					0.003^b^
Males	386	35.8	48.35	9.51	
Females	693	64.2	50.29	8.48	
**Place of birth**					0.008^b^
Pasto (capital city)	557	51.6	48.70	9.53	
Other place	522	48.4	50.56	8.09	
**Residency**					0.096^b^
Pasto (capital city)	924	85.6	49.70	9.05	
Other place	155	14.4	49.01	8.04	
**Living zone**					< 0.001^b^
Rural	139	12.9	46.29	9.11	
Urban	940	87.1	50.09	8.78	
**Socioeconomic status**					< 0.001^b^
Low	727	67.4	48.61	8.95	
Middle/High	352	32.6	51.63	8.48	
**Marital status** (wife/husband/partner)					0.048^b^
Yes	424	39.3	50.13	9.05	
No	655	60.7	49.25	8.80	
**Health insurance**					-
Yes	1.079	100.0	49.60	8.91	
No	-	-	-	-	
**Type of health insurance**					< 0.001^b^
Public	620	57.5	47.71	8.92	
Private	459	42.5	52.15	8.23	
**Education**					< 0.001^a^
None	124	11.5	48.63	7.92	
Primary	483	44.8	49.13	9.07	
Secondary	318	29.5	49.72	8.94	
University	154	14.3	51.60	8.86	
**Occupation**					< 0.001^b^
Yes	700	64.9	48.92	8.80	
No	379	35.1	50.86	8.98	
**Barriers to oral healthcare**					0.011^a^
0–1 barriers	184	17.1	50.14	8.19	
2–3 barriers	540	50.0	50.39	8.36	
≥ 4 barriers	355	32.9	48.11	9.86	
**Accessibility barriers**					< 0.001^b^
Yes	662	61.4	48.78	9.24	
No	417	38.6	50.91	8.20	
**Economic barriers**					0.755^b^
Yes	809	75.0	49.53	8.99	
No	270	25.0	49.82	8.67	
**Experience barriers**					0.236^b^
Yes	771	71.5	49.86	8.97	
No	308	28.5	48.95	8.72	

Note: GOHAI scores: values between 51–56 indicated moderate and ≤ 50 low oral health-related quality-of-life. ^a^Kruskal-Wallis and ^b^Mann Whitney tests. Significant at *P* < 0.05

Frailty was measured via CFAI, and was higher in those ≥ 85 years, those with low socioeconomic status, those without a companion, those with public health insurance, and those without education (Table 3).

**Table 3:** Means and standard deviations (SD) of comprehensive frailty assessment instrument (CFAI) dimensions by sociodemographic characteristics

Variable	*n* *(n = 1*,*079)*	CFAI dimensions mean (SD)				Total CFAI 21.09 (11.17)
		Physical 2.49 (2.92)	Psychological 6.24 (5.51)	Social 7.45 (3.09)	Environmental 4.91 (4.73)	
**Group of age** ^a^						
60–74 years	674	1.81 (2.44)	6.07 (5.57)	7.40 (3.03)	5.05 (4.90)	20.33 (11.22)
75–84 years	320	3.21 (3.20)	6.03 (4.98)	7.53 (3.09)	4.53 (4.33)	21.30 (10.35)
≥ 85 years	85	5.18 (3.12)^***^	8.41 (6.51)^**^	7.54 (3.48)	5.21 (4.81)	26.34 (12.31)^***^
**Sex** ^b^						
Males	386	1.72 (2.52)	6.01 (5.79)	7.48 (3.18)	4.01 (4.83)	19.21 (11.68)
Females	693	2.92 (3.03)^***^	6.37 (5.35)^*^	7.44 (3.04)	5.41 (4.61)^***^	22.14 (10.74)^***^
**Place of birth** ^b^						
Pasto (capital city)	557	2.51 (2.83)	6.85 (6.03)^**^	7.36 (3.18)	5.47 (5.16)^**^	22.20 (12.52)^*^
Other place	522	2.47 (3.01)	5.59 (4.83)	7.54 (2.98)	4.30 (4.16)	19.91 (9.38)
**Residency** ^b^						
Pasto (capital city)	924	2.56 (2.92)^*^	6.50 (5.62)^***^	7.39 (3.05)	5.13 (4.80)^***^	21.58 (11.43)^**^
Other place	155	2.08 (2.89)	4.70 (4.53)	7.84 (3.30)	3.59 (4.14)	18.21 (8.93)
**Living zone** ^b^						
Rural	139	1.57 (2.31)	5.60 (5.20)	8.08 (3.43)^*^	4.66 (4.78)	19.91 (10.66)
Urban	940	2.63 (2.98)^***^	6.34 (5.55)	7.36 (3.02)	4.94 (4.73)	21.27 (11.24)
**Socioeconomic status** ^b^						
Low	727	2.78 (3.01)^***^	6.65 (5.67)^***^	7.57 (3.07)^*^	5.96 (4.65)^***^	22.97 (11.23)^***^
Middle/High	352	1.89 (2.63)	5.41 (5.08)	7.20 (3.11)	2.73 (4.13)	17.22 (9.99)
**Marital status**^b^ (wife/husband/partner)						
Yes	424	1.79 (2.59)	5.42 (5.35)	8.23 (3.06)^***^	4.38 (4.71)	19.83 (11.16)
No	655	2.94 (3.03)^***^	6.77 (5.55)^***^	6.95 (3.00)	5.25 (4.72)^***^	21.91 (11.11)^***^
**Type of health insurance** ^b^						
Public	620	2.71 (2.97)^***^	6.65 (5.50)^***^	7.46 (2.98)	5.93 (4.82)^***^	22.75 (11.04)^***^
Private	459	2.19 (2.83)	5.69 (5.49)	7.43 (3.23)	3.53 (4.24)	18.85 (10.96)
**Education** ^a^						
None	124	3.84 (3.29)^***^	6.08 (4.86)	7.40 (2.87)	6.37 (4.61)^***^	23.69 (10.51)^***^
Primary	483	2.82 (3.02)	6.77 (5.77)^**^	7.65 (3.12)^**^	5.86 (4.68)	23.10 (11.48)
Secondary	318	1.93 (2.63)	6.09 (5.46)	7.49 (2.93)	4.19 (4.50)	19.71 (10.14)
University	154	1.51 (2.19)	5.03 (5.08)	6.78 (3.39)	2.23 (4.09)	15.55 (10.43)
**Occupation** ^b^						
Yes	700	2.29 (2.76)	5.88 (5.23)	7.43 (3.09)	5.44 (4.99)	21.03 (11.15)
No	379	2.87 (3.16)^**^	6.92 (5.94)^**^	7.49 (3.08)	3.93 (4.05)^***^	21.21 (11.22)
**Barriers to oral healthcare** ^a^						
0–1 barriers	184	2.38 (2.91)	6.20 (5.52)	6.82 (3.45)	4.25 (4.67)	19.65 (10.41)
2–3 barriers	540	2.63 (3.02)	5.82 (5.11)	7.32 (2.85)	4.70 (4.37)	20.47 (10.32)
≥ 4 barriers	355	2.33 (2.76)	6.91 (6.02)	7.97 (3.16)^***^	5.56 (5.22)^*^	22.78 (12.54)^*^
**Accessibility barriers** ^b^						
Yes	662	2.42 (2.83)	6.41 (5.63)	7.66 (3.01)^**^	5.08 (4.93)	21.58 (11.66)
No	417	2.61 (3.06)	5.97 (5.32)	7.12 (3.18)	4.63 (4.40)	20.32 (10.31)
**Economic barriers** ^b^						
Yes	809	2.39 (2.88)	5.75 (5.27)	7.39 (2.95)	5.06 (4.76)	20.60 (10.99)
No	270	2.79 (3.03)^*^	7.71 (5.96)^***^	7.63 (3.47)	4.45 (4.63)	22.58 (11.59)^**^
**Experience barriers** ^b^						
Yes	771	2.43 (2.88)	6.38 (5.67)	7.63 (3.13)^**^	4.87 (4.76)	21.31 (11.46)
No	308	2.65 (3.02)	5.89 (5.09)	6.99 (2.92)	5.01 (4.68)	20.54 (10.40)

Note: CFAI scores: values > 21.9 indicate mild frailty and > 38.8 high frailty. ^a^Kruskal-Wallis and ^b^Mann Whitney tests. Statistical significance at *P < 0.05, **P < 0.01, ***P < 0.001

**Fig. 3:**
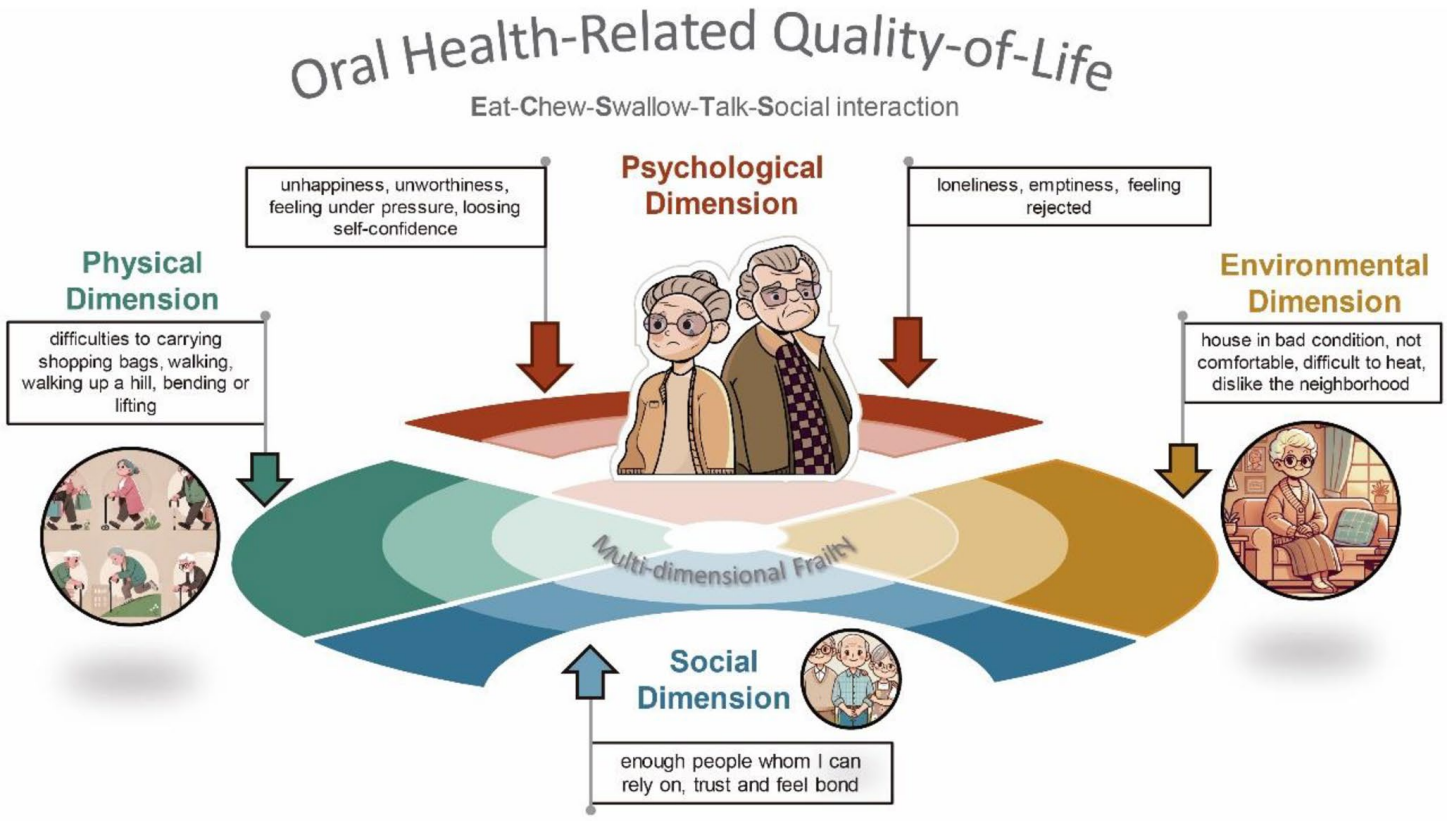
Oral health and multi-dimensional Frailty

The OHRQoL was associated with age, biological sex, living zone, type of health insurance, accessibility barriers, and comprehensive frailty, but having a worse effect on males (β= -3.06, 95% CI:-4.08– -2.03, *P* < 0.001), those living in rural zone (β= -2.45, 95% CI:-3.98– -0.92, *P* = 0.002), those who have public health insurance (β= -1.99, 95% CI:-3.00– -0.97, *P* < 0.001) and those who have accessibility barriers (β= -1.54, 95% CI:-2.51– -0.57, *P* < 0.001) (Table 4). Overall, dimensions of frailty decreased the OHRQoL, with a particular impact of the psychological dimension (β= -0.45, 95% CI: -0.54– -0.35, *P* < 0.001) on OHRQoL. However, social dimension increased the OHRQoL (β = 0.31, 95% CI: 0.15–0.46, *P* < 0.001) (Fig. 3).

**Table 4:** Association between comprehensive frailty and sociodemographic characteristics and OHRQoL (via GOHAI)

Parameter	β (GOHAI score)			
	Unadjusted [95% CI]	*P*-value	Adjusted [95% CI]	*P*-value
(Intercept)			42.53 [37.77–47.97]	< 0.001
**Age** (*continuous)*	0.14 [0.08–0.20]	< 0.001	0.17 [0.11–0.23]	< 0.001
**Biological sex**: males ref. females	-1.94 [-3.04– -0.84]	< 0.001	-2.90 [-3.90– -1.89]	< 0.001
**Residency**: Pasto (capital) ref. other place	0.69 [-0.82–2.21]	0.370	1.33 [-0.12–2.79]	0.073
**Living zone**: rural ref. urban	-3.79 [-5.36– -2.22]	< 0.001	-2.55 [-4.09– -1.01]	0.001
**Type of health insurance**: public ref. private	-4.44 [-5.48– -3.40]	< 0.001	-2.17 [-3.17– -1.17]	< 0.001
**Accessibility barriers**: yes ref. no	-2.13 [-3.21– -1.05]	< 0.001	-1.62 [-2.59– -0.66]	< 0.001
**Frailty** *(continuous)*				
Physical dimension	-0.41 [-0.59– -0.23]	< 0.001	-0.27 [-0.46– -0.09]	0.004
Psychological dimension	-0.54 [-0.63– -0.45]	< 0.001	-0.44 [-0.54– -0.34]	< 0.001
Social dimension	0.00 [-0.17–0.18]	0.964	0.33 [0.17–0.48]	< 0.001
Environmental dimension	-0.58 [-0.68 - -0.47]	< 0.001	-0.34 [-0.45 - -0.22]	< 0.001
Scale			59.37 [54.56–64.59]	

Note: A General Linear Model was estimated with age, biological sex, residency, living zone, type of health insurance, accessibility barriers, and frailty dimensions via CFAI (physical, psychological, social, and environmental) and OHRQoL (via GOHAI)

## Discussion

Using a cross-sectional design, we found significant associations between comprehensive frailty dimensions and OHRQoL, assessed via GOHAI. Our model showed that being male, living in a rural zone, having public health insurance as well as accessibility barriers negatively impacted OHRQoL, as did frailty, with the psychological dimension having a higher negative than other dimensions, while the social dimension increased OHRQoL.

Overall, the definition of quality of life implies well-being, which is a subjective construct, usually based on self-worth, self-confidence, coping strategies, and the control of emotions. In this study, frailty experienced via psychological dimension (feelings of unhappiness, symptoms of depression, non-self-confidence, loneliness, or emptiness) was associated with OHRQoL. Our finding is in line with other research showing that psychological aspects, in this case depression, significantly worsened OHRQoL over time [35]. Similarly, loneliness has been shown to impact health and quality of life [36]. Vice versa, OHRQoL was positively associated with social skills, likely because it co-determines the way one interacts with the world and others.

The mean of OHRQoL was low (49.60, SD = 8.9) in our study, and was similar to that yielded in a study on older Mexicans (48.07, SD = 0.01) [37], higher than that those patients with rheumatoid arthritis from Iran (32.9, SD = 4.8) [38] and lower than that from older populations from Sudan (53.96 SD = 6.31) [39] or China (54.95, SD = 6.47) [40]. OHRQoL differed significantly between sexes, with men having a lower score (worse OHRQoL) than women. Interestingly, such sex difference has not been frequently reported before [41–43]. It is possible that men overall had worse dental conditions in our sample, something which we were unable to ascertain. Additionally, there were significant variations of OHRQoL by SES, with low SES being associated with lower OHRQoL. This has been found before, also for South American countries [44] and likely linked to low SES also meaning lower accessibility to oral healthcare due to financial reasons.

We also found living rurally comes with lower OHRQoL. Geographic differences in OHRQoL have been reported before, likely because of access to care, but also concordantly better economic situations [45]. Similarly, associations with education have been found before, with education being closely linked to financial means, but also literacy, i.e. awareness and understanding of oral health and the ability to seek care if needed [46]. Generally, and understandably, OHRQoL was better for individuals who did not report relevant accessibility barriers to oral healthcare, which points again to the relevance of care accessibility (physical, intellectual or financial) as relevant factor.

Most relevant for the present study, a higher score of frailty was associated with lower OHRQoL, also when adjusting for the discussed other aspects impacting quality of life. Again, this association has been reported before – albeit not always in a consistent direction for populations in Mexico [47] or Japan [48], while in these reports, the focus was on physical frailty. In our study, the psychological dimension of frailty showed the strongest association with OHRQoL. Using a multidimensional instrument allowed us to reflect on the complex web of factors contributing to frailty and draw a more complete and nuanced understanding of the association between frailty and OHRQoL. Such holistic understanding is crucial for developing comprehensive interventions addressing frailty for improving OHRQoL in older populations.

### Limitations

While cross-sectional studies offer a range of advantages over alternative designs (e.g. being relatively less resource-intensive and allowing a prospective survey design), they are prone to bias and cannot demonstrate causal relationships, mainly as cause-effect observations are impossible given that only one time point is assessed. Employing longitudinal studies to assess the associations between risk factors and disease development would allow us to observe the progression of health events and their connections over different time periods. Our sample was non-probabilistic which may affect generalizability of our results. Moreover, self-reports, such as the ones used by our study. are known to be associated with desirability bias, something we cannot exclude or control for. Additionally, no dental examination was performed, while we assume that the dental status is a significant driver of OHRQoL. Future studies should strive to include such examination, allowing them to draw a more comprehensive picture of OHRQoL and relevant factors impacting on it.

## Conclusions

Comprehensive frailty assessment allows us to map various dimensions of frailty and their impact on OHRQoL. In the present study, psychological aspects of frailty were linked to OHRQoL, among other (well established) factors determining OHRQoL. Oral health policies in older populations or individuals should consider such psychological aspects further, and research around OHRQoL in older groups should aim to comprehensively characterize frailty. Collaboration between dental professionals and psychologists to integrate oral health into overall mental health assessments and treatment plans is recommended.

## Electronic supplementary material

Below is the link to the electronic supplementary material.

Supplementary Material 1

## Data Availability

Derived data supporting the analyses of this study are available online as supplemental material.
